# Dietary Glycemic Index, Dietary Glycemic Load, Blood Lipids, and Coronary Heart Disease

**DOI:** 10.1155/2010/170680

**Published:** 2010-02-28

**Authors:** Edgar Denova-Gutiérrez, Gerardo Huitrón-Bravo, Juan O. Talavera, Susana Castañón, Katia Gallegos-Carrillo, Yvonne Flores, Jorge Salmerón

**Affiliations:** ^1^Centro de Investigación en Ciencias Médicas, Universidad Autónoma del Estado de México, 50130 Toluca Estado de México, Mexico; ^2^Unidad de Investigación Médica en Epidemiología Clínica, Hospital de Especialidades CMN SXXI, 06720 México, Mexico; ^3^Unidad de Investigación Epidemiológica y en Servicios de Salud, Instituto Mexicano del Seguro Social, 65450 Cuernavaca Morelos, Mexico

## Abstract

*Objective*. To examine the associations of dietary glycemic index (GI) and dietary glycemic load (GL) with blood lipid concentrations and coronary heart disease (CHD) in nondiabetic participants in the Health Worker Cohort Study (HWCS). *Materials and Methods*. A cross-sectional analysis was performed, using data from adults who participated in the HWCS baseline assessment. We collected information on participants' socio-demographic conditions, dietary patterns and physical activity via self-administered questionnaires. Dietary GI and dietary GL were measured using a validated food frequency questionnaire. Anthropometric and clinical measurements were assessed with standardized procedures. CHD risk was estimated according to the sex-specific Framingham prediction algorithms. *Results*. IIn the 5,830 individuals aged 20 to 70 who were evaluated, dietary GI and GL were significantly associated with HDL-C, LDL-C, LDL-C/HDL-C ratio, and triglycerides serum levels. Subjects with high dietary GI have a relative risk of 1.56 (CI 95%; 1.13–2.14), and those with high dietary GL have a relative risk of 2.64 (CI 95%; 1.15–6.58) of having an elevated CHD risk than those who had low dietary GI and GL. *Conclusions*. Our results suggest that high dietary GI and dietary GL could have an unfavorable effect on serum lipid levels, which are in turn associated with a higher CHD risk.

## 1. Introduction

Coronary Heart Disease (CHD) is a major public health problem and the leading cause of death in Mexico [1]. Considerable epidemiological evidence shows that lifestyle, especially diet, influences occurrence of CHD. The role that dietary carbohydrates play in CHD risk has recently received particular attention [2, 3]. High carbohydrate intake has been found to have an adverse effect on serum lipid levels and glucose metabolism, which are likely to increase the risk of CHD [4–6].

Glycemic index (GI) and glycemic load (GL) have been used to quantify the glycemic burden of carbohydrates in particular foods. The GI is the average propensity of carbohydrates in the diet to increase blood glucose compared with a reference food [7]. The GL is defined as the product of GI and the carbohydrate content, reflecting both a food's glycemic index and carbohydrate levels [8, 9]. Both dietary GI and GL appear to have increased in recent years because of increasing carbohydrate intake and changes in food processing [10]. Diets with a high GI and GL are positively associated with CHD [11], and type 2 diabetes [8, 9], probably in part because they have adverse effects on blood lipid levels; some metabolic studies have observed that diets with a high GI or GL lead to an increase in triglycerides and reduced high density lipoprotein cholesterol (HDL-C) [4–6, 12–14] and cause systemic inflammation [15, 16].

In order to address the hypothesis that diets that cause a high glycemic response increase the risk of a high lipid profile and CHD, we examined the associations of dietary GI and dietary GL with blood lipid levels and CHD risk among Health Worker Cohort Study (HWCS) participants.

## 2. Material and Methods

### 2.1. Study Population

We performed a cross-sectional analysis of data from adults participating in the baseline assessment of the Health Workers Cohort Study (HWCS). The study design, methodology, and participants' baseline characteristics have been detailed elsewhere [17–19]. Briefly, this cohort study focuses on lifestyle and chronic disease. Between March 2004 and April 2006, 9,467 employees and their relatives from three different health and academic institutions in Morelos and the Mexico states in Mexico were invited to participate in the study, and 8,307 apparently healthy adults were formally enrolled. Participants completed baseline questionnaires providing information about demographic, behavioral, and lifestyle factors; medical history including medication use; height and weight; and use of multivitamins and other supplements.

In the present analysis, we included 5,830 subjects aged 20–70 who provided fasting blood samples (≥8 hours since last meal), and were not previously diagnosed with type 2 diabetes (assessed by self-report or with levels of glucose ≥126 mg/dL) or taking lipid-lowering medication. We also excluded participants who left 10 or more food items blank on the questionnaire (*n* = 695), and who did not consume between 600 kcal and 7000 kcal (*n* = 197) daily.

The ethics committees of all participating institutions reviewed and approved the study protocol.

### 2.2. Data Collection

#### 2.2.1. Assessment of Nondietary Variables

Demographic characteristics were evaluated from data reported on self-administered questionnaires. Physical activity was assessed using the International Physical Activity Questionnaire (IPAQ) instrument [20, 21], which was adapted for the Mexican population. Participants were asked about their leisure activity, recreational activity, daily activity, and any physical labor associated with employment. The IPAQ section on leisure time activity included 16 items on the amounts of weekly time spent performing exercises like walking, running, and cycling. Each activity was given a value in metabolic equivalents (Mets) and total Mets per week were calculated.

Participants were also asked about the weight changes they had experienced within the previous year. This information was categorized as: no weight change, weight lost, or weight gained in the previous year (less/more than 5 kg).

#### 2.2.2. Anthropometric and Clinical Assessment

Weight was measured after an overnight fasting with a previously calibrated electronic TANITA scale (model BC-533; Tokyo, Japan), with participants, who had fasted overnight, wearing minimal clothing. Height was measured using a conventional stadiometer while the subjects were standing, barefoot, with their shoulders in a normal position. Waist circumference was measured to the nearest 0.1 cm at the high point of the iliac crest at the end of normal expiration, with a steel measuring tape, which was placed below any clothing, directly touching the participant's skin. Body mass index (BMI kg/m^2^) was calculated as a ratio of weight (kg) to height squared (m^2^). Proportion of body fat was estimated with the reference technique, using dual-energy X-ray absorptiometry (DEXA) performed with a Lunar DPXL whole body X-ray densitometer (Lunar Radiation Corp., Madison, WI; software version 1.35, fast scan mode; model: DPX-NT 73735, series: 638405U77).

Blood pressure was measured with an automatic digital blood pressure monitor. Participants were seated with their right arm resting at heart level. All measurement procedures were performed by nurses trained to use standardized procedures (reproducibility was evaluated, resulting in concordance coefficients between 0.83–0.90).

#### 2.2.3. Dietary Assessment

A semiquantitative food frequency questionnaire (FFQ) validated in a Mexican population [22], where a correlation coefficient *r* = 0.50; energy-adjusted between instruments for carbohydrates was observed and was used to assess diet. This questionnaire included data on frequency of consumption of 116 food items during the previous year. For each food, a commonly used portion size (e.g., 1 slice of bread) was specified on the FFQ, and the participants were asked how frequently they had consumed the food over the previous year. They chose from ten responses, ranging from “never” and “less than once per month” to “6 or more times per day.” We estimated the energy and nutrient intake by multiplying the frequency of consumption of each food by the nutrient content, estimated with a comprehensive database of food contents [23].

The methods used to assess the GI and GL have been described previously [8, 9, 11]. For foods included on the FFQ, we used a published international table of GI values [24]. The average GI of the individual's diet, an indicator of the overall quality of their carbohydrate intake, was determined using the following formula: dietary glycemic index = ∑_foods_C  × F × GI/∑_foods_C  × F; where C represents the grams of carbohydrate in a portion of food, F the frequency of consumption of the food, and GI the GI using glucose as the reference. The GL of each food was computed by multiplying the food's GI and the carbohydrate content of one food portion (glycemic load = C × F × GI/100 [8, 9]). The dietary GL for each participant was calculated by multiplying the GL per portion of food by the frequency of consumption. The sum of these products over all food items was the dietary GL. The nutrients, dietary GI, and dietary GL were energy-adjusted using the residuals method [25].

#### 2.2.4. Biomarkers Assessment

A fasting venous blood sample (fasting time was ≥8 hours, to remain consistent with previous analyses of data from adults participating in the National Health and Nutrition Examination Survey (NHANES) [6]) was collected from each participant. Plasma triglycerides were measured with a colorimetric method following enzymatic hydrolysis performed with the lipase technique. High-density lipoprotein cholesterol (HDL-C) was measured by clearance method; in this method non HDL-C lipoprotein is removed in the first step of the reaction (clearance step); this method shows excellent correlation with the reference method (*r* = 0.99). low-density lipoprotein cholesterol (LDL-C) was measured by clearance method; correlation studies on the LDL clearance method produced a coefficient of *r* = 0.985 with ultracentrifugation; total cholesterol was measured by colorimetric method following enzymatic assay. All biomedical assays were performed using a Selectra XL instrument (Randox), in concordance with the proceedings of the International Federation of Clinical Chemistry and Laboratory Medicine [26]. Furthermore, we calculated the ratio of LDL-C to HDL-C.

#### 2.2.5. High Lipid Profile and Risk of Coronary Heart Disease

High lipid profile was defined according to the criteria put forth in the report of the National Cholesterol Education Program (NCEP) ATP-III, that defines a high lipid profile as: high serum triglycerides ≥ 150 mg/dL, high serum total cholesterol ≥ 200 mg/dL, high LDL-C ≥ 100 mg/dL, and low HDL-C < 40 mg/dL in men and <50 mg/dL in women [27].

Coronary heart disease (CHD) risk was calculated using a recalibration of the Framingham coronary heart disease proposed by D'Agostino et al. [28, 29]. Sex-specific prediction algorithms accounted for age, cigarette smoking, blood pressure measurement, and levels of HDL-C and LDL-C. We defined participants as at no CHD risk when they had less than 10 percent risk in 10 years. Subjects who had more than 10 percent risk in ten years were defined as having a CHD risk (moderate/elevated) by American Heart Association standards [30].

#### 2.2.6. Statistical Analysis

We performed a descriptive analysis of the characteristics of interest using quartiles of the natural distribution of the dietary GI and GL estimates. Linear regression was used to calculate *P* values for continuous variables, and *χ* ^2^ tests were used for categorical variables. We computed the means for total cholesterol, triglycerides, LDL-C, and HDL-C and LDL-C/HDL-C ratio by quartiles of dietary GI and GL, and we adjusted these means by age and sex.

We examined the influence of dietary GI and GL on lipid profile using multivariate regression models with continuous variables. To estimate the magnitude of the association between specific categories of dietary GI or GL and lipid profile, as well as elevated CHD risk, we computed adjusted odds ratios (OR) and 95% CI with multiple logistic regression models. All *P* values presented are 2-tailed, and a *P* < .05 was considered statistically significant. All statistical analyses were performed using STATA software, version 9.0 [31].

## 3. Results

This analysis included data from a sample of 5,830 subjects, 71.9% of whom were female. Their average dietary GI was 73 (SD = 17), and their mean dietary GL was 245 (SD = 127). For the total study population, soft drinks, white bread, pastries, and cookies were the main contributors to the GL, contributing 21.0%, 13.6%, 9.4%, and 7.5%, respectively. Dietary GI and dietary GL were moderately correlated (*r* = 0.52, *P* < .001). Dietary GL was positively associated with carbohydrate intake (*r* = 0.88, *P* < .001), and dietary GI and carbohydrate intake were also correlated, though less so (*r* = 0.11, *P* < .001) (data not shown). Subjects with high dietary GI tended to have a higher proportion of body fat and to have lower intake of magnesium, fiber, and cholesterol than subjects with low dietary GI. Subjects with high dietary GL tended to be more physically active, in addition, they consumed less energy derived from protein and fat, and higher average folate, magnesium, fiber, cholesterol, and alcohol intake compared with subjects in the lowest quartile of dietary GL (Table 1).

The blood plasma concentrations of total cholesterol, HDL-C, LDL-C, triglycerides, and the LDL-C/HDL-C ratio by quartiles of dietary GI and GL are shown in Table 2. For the lowest and the highest quartiles of GI, the age and sex-adjusted mean HDL-C blood plasma concentrations were 40.5 and 38.8 mg/dL (*P* = .001); LDL-C blood plasma concentrations were 114.0 and 118.0 mg/dL (*P* = .03); triglycerides were 152.4 and 158.2 mg/dL (*P* = .02); LDL-C/HDL-C ratios were 3.0 and 3.2 (*P* < .001), respectively. Higher dietary GL was associated with a small decrease in HDL-C and with increases in triglycerides. However, in some cases the differences in the lipid concentrations between quartiles were small and appear not to be statistically significant.

Table 3 shows the effects of dietary GI and GL on lipid profile. After adjusting for demographic characteristics, life style factors, and consumption of energy, saturated fat, polyunsaturated fat, trans fat, folate, magnesium, fiber, and alcohol, we observed that participants in the highest quartile of dietary GI showed an increase of 3.1 mg/dL in total cholesterol (*P* = .04); 4.8 mg/dL in LDL-C (*P* < .01), 5.8 mg/dL in triglycerides (*P* = .01). Furthermore, we observed that subjects with higher dietary GI presented a decrease of 1.9 mg/dL of HDL-C (*P* = .001) compared with subjects in the lower dietary GI category. Similarly, participants within the highest quartile of dietary GL had an increase of 8.2 mg/dL in total cholesterol (*P* = .01), 6.0 mg/dL in LDL-C (*P* = .002), 17.7 mg/dL in triglycerides (*P* = .01). Subjects with high dietary GL also showed a decrease of 3.5 mg/dL in HDL-C (*P* = .001) compared with subjects in the lower quartile of dietary GL.

Table 4 indicates that subjects in the highest quartile of dietary GI distribution have a greater risk of a low HDL-C (OR = 1.32; CI 95%, 1.07–1.62), a greater risk of having high LDL-C (OR = 1.26; CI 95%, 1.02–1.55), and a greater risk of having high triglycerides (OR = 1.18; CI 95%, 1.01–1.40) than those in the lowest dietary GI quartile. We also observed that subjects in the upper quartile of dietary GL have a greater risk of low HDL-C (OR = 1.78; CI 95%, 1.19–2.57) and greater risk of high triglycerides (OR = 1.85; CI 95%, 1.12–3.04) than those on the lowest quartile of dietary GL.

Additionally, subjects in the upper quartile of dietary GI have a 56% greater risk of an elevated 10-year CHD estimate than those in the lowest quartile. Similarly, subjects in the highest quartile of the dietary GL distribution have an OR = 2.64 (CI 95%; 1.15–6.58) of presenting CHD risk estimate compared to those in the lowest dietary GL quartile (Table 4).

## 4. Discussion

Our results suggest that diets with a high GI and high GL were positively associated with blood lipid levels that posed an elevated CHD risk, independent of other known risk factors. Our results indicate that in our study population, dietary GL may be a more accurate predictor of lipid profile and CHD risk than dietary GI, because this measure captures the combined metabolic and health effects of the quality and quantity of carbohydrate consumption [11–15], although it may not be the same among others. In our population, the average blood plasma concentrations of triglycerides, LDL-C, and LDL-C/HDL-C ratio increased across the quartiles of dietary GI, while triglycerides and the LDL-C/HDL-C ratio increased across dietary GL categories. Additionally, we found that blood plasma concentrations of HDL-C decreased as dietary GI or GL increased. 

Our analysis shows that subjects in the higher dietary GI group have a 1.32 times greater risk of low HDL-C levels, a 1.26 times greater risk of elevated LDL-C blood plasma concentrations, and 1.18 times greater risk of high triglycerides than those in the lower dietary GI quartile. We observed that subjects in the upper quartile of the dietary GL have a 1.78 times greater risk of low HDL-C levels, and a 1.85 times greater risk of high triglycerides than those in the lowest quartile. Other studies have suggested that a high dietary GI or high dietary GL can have detrimental effects on lipid metabolism [4, 15, 16]. In most studies, high dietary GI or dietary GL has been associated with higher triglyceride blood plasma concentrations [4, 14], and lower HDL-C levels [4–6, 12–14, 32–35]. In our population, we observed a high level of triglycerides (152.6 mg/dL) and low levels of HDL-C (39.8 mg/dL). Furthermore, our analyses show that subjects in the highest quartile of dietary GL would entail a 13.7–17.7 mg/dL excess of triglycerides and a deficit of 3.5 mg/dL of HDL-C, compared to the average levels of the population. Contrary to our results, a cross-sectional study has not found a significant association between increased dietary GI or dietary GL and higher levels of LDL-C [5], perhaps because this study used inadequate sample size to detect an association.

In our population, subjects with a high dietary GI present a 1.56 times greater risk of CHD than those with a low dietary GI. Subjects in the upper quartile of dietary GL have an almost threefold greater risk of CHD estimate [OR = 2.64; CI 95%; 1.15–6.58] than those in the lowest quartile. These results support the findings of previous investigations [11–15, 33, 36] which suggest an increased risk of cardiovascular disease; for example, a meta-analysis by Barclay et al. provides evidence that diets with a high GI increase the risk of heart disease (RR = 1.25; CI 95%, 1.00–1.56). Other studies also support the hypothesis that a low GI and/or low GL diet has an important effect on lipid markers and CHD risk reduction [37–39].

Mechanisms have been proposed to explain the association of a high GI or high GL diet on high blood lipid concentrations and CHD risk. There is strong evidence that diets with a high GI or GL may directly increase insulin resistance through their effects on glycemia, free fatty acids, and counter regulatory hormone secretion [10, 40]. Insulin resistance seems to cause increases in triglycerides and decreases in HDL-C [10, 41]. High glucose and insulin concentrations are associated with increased risk profiles for CHD, including increased glycosylated proteins, oxidative status, hemostatic variables, and poor endothelial function [40, 41]. The present study's cross-sectional design makes it difficult to examine the causal relationship between dietary GI or GL and the concentrations of blood lipids or CHD risk since the temporal relation of these events may not be clearly established. Furthermore, this might be a result of a possible report bias and cannot be interpreted as a causal relationship. The participants in this cohort study are adults from a specific segment of the Mexican population: working class, seemingly healthy individuals. While these adults cannot be considered representative of the Mexican adult population as a whole, they may be considered representative of middle to low income adults residing in the urban areas of central Mexico. Despite these limitations, our results provide relevant information about the association between dietary GI and dietary GL and lipids profile, as well as 10-year CHD risk estimates in our population.

## 5. Conclusion

Our findings suggest that both the quality and the quantity of carbohydrate consumption significantly influence blood lipid concentrations and CHD risk in Mexican adults free of diabetes and lipid disorders. Our data strongly support the hypothesis that diets with a low GI and GL—which include foods like whole grains (e.g., whole grain breads, barley and wheat germ), vegetables, legumes, fruits, and nuts—are associated with a more favorable lipid profile that may be cardioprotective.

## Figures and Tables

**Table 1:** Characteristics of participants in the Health Worker Cohort Study by quartiles of dietary glycemic index and dietary glycemic load.

	Quartiles of dietary glycemic index					Quartiles of dietary glycemic load				
	Q1 (55)^1^	Q2 (67)	Q3 (77)	Q4 (94)	*P*-value^§^	Q1 (63)^1^	Q2 (158)	Q3 (296)	Q4 (525)	*P*-value^§^
	(*n* = 1458)^2^	(*n* = 1457)	(*n* = 1458)	(*n* = 1457)		(*n* = 1458)^2^	(*n* = 1457)	(*n* = 1458)	(*n* = 1457)	
*Sex (women*)^€^	74.4	70.6	72.7	69.8	.03	74.3	74.2	69.6	67.6	.001

*Age (years)* ^†^	40.9	40.2	40.0	39.2	<.01	39.4	40.5	39.9	38.7	.01

*BMI (kg/m)* ^‡^	26.6	26.8	26.5	26.9	.01	26.9	26.6	26.7	27.3	.04

*Body fat proportion* (% *of fat*)^†^	30.9	30.7	31.0	31.4	.1	30.9	31.0	31.0	31.1	.2

*Waist Circumference (cm)* ^†^	89.0	89.4	89.2	89.9	.001	89.6	89.1	89.5	90.1	.05

*Physical Activity (mets/week)* ^†^	120.4	124.8	124.8	121.7	.8	114.5	118.5	126.6	136.5	<.001

*Smoking (*%)^€^										
Current	16.5	18.6	21.2	24.7	<.001	25.2	19.4	21.5	18.2	.3
Past	24.1	23.3	24.4	24.5		15.8	23.6	24.6	27.2	
Never	59.4	58.1	54.4	50.8		58.0	57.0	53.9	54.6	

*Multivitamin Use (*%)^€^										
Current	7.3	6.5	6.0	5.3	.1	4.1	6.3	6.6	5.6	.1
Past	24.2	24.1	25.7	23.7		20.5	24.0	24.8	26.6	
Never	68.5	69.4	68.3	71.0		75.4	69.7	68.6	67.8	

*Nutrient Intake* ^†^										
Total energy (kcal/day)*	2093	2218	2252	2171	<.01	1000	1701	2561	3767	<.001
Carbohydrate (% energy)*	57.9	58.9	61.1	63.6	<.001	50.4	57.9	62.7	67.9	<.001
Protein (% energy)*	22.5	22.0	21.1	19.8	<.001	27.5	22.7	19.9	17.5	<.001
Saturated fat (% energy)*	9.3	9.4	8.9	8.3	<.001	10.8	9.4	8.6	7.6	<.001
Trans fat (% energy)*	0.53	0.49	0.44	0.39	<.001	0.48	0.47	0.45	0.42	<.001
Monounsaturated fat (% energy)*	10.6	10.4	9.9	9.3	<.001	12.1	10.5	9.5	8.7	<.002
Polyunsaturated fat (% energy)*	4.6	4.4	4.1	3.9	<.001	5.0	4.5	4.0	3.8	<.001
Cholesterol (mg/day)*	245	259	248	239	.08	164	215	274	350	<.001
Fiber (mg/day)*	18.5	17.7	17.0	14.4	<.001	7.9	13.7	19.1	29.0	<.001
Magnesium (mg/day)*	374	386	385	348	<.001	184	298	429	639	<.001
Folate (mg/day)*	372	404	411	379	.3	171	305	449	729	<.001
Alcohol (g/day)*	2.7	3.7	4.8	4.3	<.001	2.2	3.3	4.4	5.6	<.001

^§^ *P* values from linear regression (continuous variables) or chi2 test (categorical variables); ^1^Median of dietary glycemic index and dietary glycemic load by quartile of consumption; ^2^Sample size by quartile of dietary glycemic index and dietary glycemic load; ^†^Means; ^€^percentages; *Energy-adjusted using the residual method; ^‡^BMI, body mass index.

**Table 2:** Mean concentrations of blood lipids by quartiles of dietary glycemic index and dietary glycemic load.

	Quartiles of dietary glycemic index					Quartiles of dietary glycemic load				
	Q1	Q2	Q3	Q4	*P* for trend	Q1	Q2	Q3	Q4	*P* for trend
*Total cholesterol (mg/dL)*										
Age and sex-adjusted^‡^	190.5 (38.8)	191.5 (38.3)	192.3 (39.1)	193.4 (39.5)	.1	188.5 (38.5)	191.7 (38.9)	193.1 (39.3)	189.0 (38.9)	.07

*HDL cholesterol (mg/dL)*										
Age and sex-adjusted^‡^	40.5 (10.7)	40 (10.4)	39.7 (10.9)	38.8 (10.0)	.001	40.3 (11.7)	40.1 (10.2)	39.6 (9.9)	38.9 (10.1)	.03

*LDL cholesterol (mg/dL)*										
Age and sex-adjusted^‡^	114.0 (36.2)	115.8 (37.0)	116.3 (37.8)	118.0 (39.4)	.03	114.4 (36.8)	115.3 (37.1)	117.4 (36.7)	114.4 (39.9)	.1

*Triglycerides (mg/dL)*										
Age and sex-adjusted^‡^	152.4 (109.1)	147.7 (80.7)	152.4 (90.9)	158.2 (87.5)	.02	144.9 (103.8)	151.7 (95.8)	153.8 (83.8)	155.8 (86.8)	<.001

*LDL/HDL cholesterol ratio*										
Age and sex-adjusted^‡^	3.00 (1.2)	3.09 (1.3)	3.11 (1.2)	3.22 (1.3)	<.001	3.02 (1.4)	3.07 (1.2)	3.14 (1.2)	3.14 (1.3)	.2

^‡^Means (SD).

**Table 3:** Mean changes of concentrations of blood lipids and CHD risk according to dietary glycemic index and dietary glycemic load.

	Quartiles of dietary glycemic index					Quartiles of dietary glycemic load				
	Q2	Q3	Q4	*P* for trend	Q1	Q2	Q3	Q4	*P* for trend
Total cholesterol	0.0	0.9	1.7	3.1	.04	0.0	4.5	5.3	8.2	.01
(mg/dL) *f *		[−1.9, 3.5]	(−0.8, 4.6)	(−0.05, 5.4)			[−1.8, 10.2]	[−1.2, 10.8]	[1.3, 14.9]	

HDL-C (mg/dL) *f *	0.0	−0.7	−1.0	−1.9	.001	0.0	−1.3	−2.1	−3.5	.001
		[−1.4, 0.1 ]	[−1.9, -0.3]	[−2.8, −1.1]			[−2.1, −0.5]	[−3.0, -1.1]	[−4.9, −1.9]	

LDL-C (mg/dL) *f *	0.0	2.2	2.9	4.8	.001	0.0	1.2	5.3	6.0	.002
		[−0.6, 4.7]	[0.1, 5.6]	[2.0, 7.7]			[−1.6, 4.0]	[2.0, 8.5]	[0.8, 11.1]	

Triglycerides (mg/dL) *f *	0.0	0.7	5.0	5.8	.01	0.0	10.0	13.7	17.7	.01
		[−3.8, 5.2]	[0.5, 9.4]	[1.1, 10.8]			[−5.0, 23.1]	[−2.2, 27.8]	[1.8, 34.1]	

LDL-C/HDL-C ratio *f *	0.0	0.1	0.16	0.29	<.001	0.0	0.17	0.39	0.59	<.001
		[0.03, 0.21]	[0.07, 0.26]	[0.19, 0.40]			[0.1, 0.26]	[0.28, 0.51]	[0.31, 0.79]	

CHD risk *f *	0.0	0.13	0.17	0.32	<.001	0.0	0.10	0.43	0.69	<.001
		[0.0, 0.25]	[0.04, 0.30]	[0.2, 0.5]			[0.0, 0.21]	[0.18, 0.67]	[0.47, 0.90]	

CHD; Coronary heart disease risk;* f* *β*'s adjusted for sex; age (years); BMI (<20, 20–24.9, 25.0–29.9, >30 kg/m2); physical activity (mets/week); smoking (never, past, current); and intakes of total energy, protein, saturated fat, polyunsaturated fat, trans fat, cholesterol, alcohol, fiber, folate, magnesium.

**Table 4:** Odds ratio of high lipids profile according to quartiles of dietary glycemic index and dietary glycemic load.

	Quartiles of dietary glycemic index					Quartiles of dietary glycemic load				
	Q1	Q2	Q3	Q4		Q1	Q2	Q3	Q4	
	OR	OR	OR	OR	*P* for trend	OR	OR	OR	OR	*P* for trend
		(CI 95%)	(CI 95%)	(CI 95%)			(CI 95%)	(CI 95%)	(CI 95%)	

High total cholesterol *f *	1.0	1.00	1.05	1.10	.1	1.0	1.17	1.28	1.24	.2
		(0.82–1.11)	(0.90–1.24)	(0.93–1.30)			(0.83–1.66)	(0.88–1.89)	(0.76–2.04)	

n**	550	550	558	562		525	549	576	551	

Low HDL-C *f *	1.0	1.13	1.30	1.32	.002	1.0	1.33	1.47	1.78	.002
		(0.94–1.36)	(1.08–1.58)	(1.07–1.62)			(1.10–1.54)	(1.17–1.87)	(1.19–2.57)	

n**	1125	1142	1170	1168		1136	1150	1166	1187	

High LDL-C *f *	1.0	1.18	1.22	1.26	.05	1.0	1.06	1.31	1.31	.05
		(0.96–1.45)	(0.98–1.53)	(1.02–1.55)			(0.74–1.50)	(0.89–1.96)	(0.77–2.22)	

n**	941	958	964	970		941	942	967	995	

High triglycerides *f *	1.0	1.00	1.03	1.18	.1	1.0	1.50	1.68	1.85	.01
		(0.82–1.12)	(0.88–1.20)	(1.01–1.40)			(1.04–2.17)	(1.13–2.49)	(1.12–3.04)	

n**	555	555	563	630		536	579	603	616	

Risk of CHD *f *	1.0	1.18	1.23	1.56	.01	1.0	1.60	1.92	2.64	.001
		(0.86–1.61)	(0.91–1.65)	(1.13–2.14)			(0.80–3.21)	(0.90–4.11)	(1.15–6.58)	

n**	37	38	41	58		28	46	48	52

High lipids profile: High total Cholesterol (≥200 mg/dL); Low HDL-C (<40 mg/dL for men and <50 mg/dL in women); High LDL-C (≥100 mg/dL); High triglycerides (≥150 mg/dL); CHD; Coronary heart disease risk (≥10 in 10 years );* f* OR's adjusted for sex; age (years); BMI (<20, 20–24.9, 25.0–29.9, >30 kg/m^2^); physical activity (mets/week); smoking (never, past, current); and intakes of total energy, protein, saturated fat, polyunsaturated fat, trans fat, cholesterol, alcohol, fiber, folate, magnesium. **n, subjects with abnormal lipid blood plasma concentrations by quartile of GI and GL consumption.
